# Unveiling Fast Field Oscillations through Comodulation

**DOI:** 10.1523/ENEURO.0079-17.2017

**Published:** 2017-08-04

**Authors:** Robson Scheffer-Teixeira, Adriano B. L. Tort

**Affiliations:** Brain Institute, Federal University of Rio Grande Do Norte, Natal, RN 59056-450, Brazil

**Keywords:** cross-frequency coupling, gamma, hipppocampus, LFP, oscillations, theta

## Abstract

Phase-amplitude coupling analysis shows that theta phase modulates oscillatory activity not only within the traditional gamma band (30–100 Hz) but also at faster frequencies, called high-frequency oscillations (HFOs; 120–160 Hz). To date, however, theta-associated HFOs have been reported by only a small number of laboratories. Here we characterized coupling patterns during active waking (aWk) and rapid eye movement (REM) sleep in local field potentials (LFPs) from the parietal cortex and hippocampus of rats, focusing on how theta-associated HFOs can be detected. We found that electrode geometry and impedance only mildly influence HFO detection, whereas recording location and behavioral state are main factors. HFOs were most prominent in parietal cortex and during REM sleep, although they could also be detected in stratum oriens-alveus and during aWK. The underreporting of HFOs may thus be a result of higher prevalence of recordings from the pyramidal cell layer. However, at this layer, spike-leaked HFOs (SLHFOs) dominate, which represent spike contamination of the LFP and not genuine oscillations. In contrast to HFOs, high-gamma (HG; 60–100 Hz) coupled to theta below the pyramidal cell layer; theta–HG coupling increased during REM sleep. Theta also weakly modulated low-gamma (LG; 30–60 Hz) amplitude, mainly in the parietal cortex; theta–LG coupling did not vary between aWK and REM sleep. HG and HFOs were maximal near the theta peak, parietal LG at the ascending phase, hippocampal LG at the descending phase, and SLHFOs at the trough. Our results unveil four types of fast LFP activity coupled to theta and outline how to detect theta-associated HFOs.

## Visual Abstract

**Figure:**
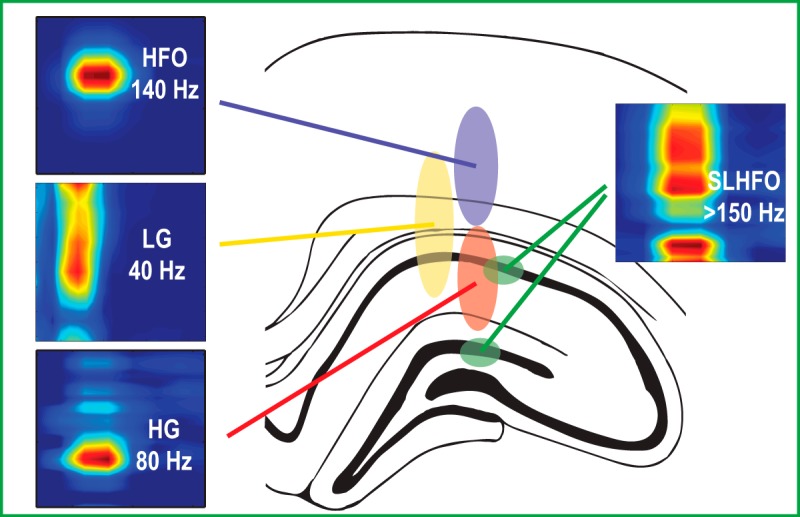

## Significance Statement

Cortical networks display theta (5–10 Hz) and gamma (30–100 Hz) oscillations, which often interact by phase-amplitude coupling. However, comodulation analysis has also shown that theta modulates oscillations at higher frequencies (120–160 Hz), which have been called HFOs. In contrast to gamma, however, knowledge about these HFOs remains scarce, since they have been found by only a few laboratories. The present study reveals how to detect theta-associated HFOs and further highlights their distinctions from gamma oscillations. Moreover, the results also distinguish theta-associated HFOs from spurious LFP activity coupled to theta that is due to spike “scars.” Altogether, comodulation analysis unveils four different types of fast LFP activity coupled to theta: two oscillations within the traditional gamma band, HFOs, and spike-leaked activity.

## Introduction

Theta waves (5–10 Hz) dominate electrophysiological activity in the rodent hippocampus and entorhinal cortex during exploratory behaviors and rapid eye movement (REM) sleep (Vanderwolf, 1969; Whishaw and Vanderwolf, 1973). Theta has been linked to both navigation and mnemonic function, including spatial and working memory (O’Keefe, 1976; O’Keefe and Nadel, 1979; Bliss and Collingridge, 1993; Lisman and Idiart, 1995; Redish, 1999). In addition, low-gamma (LG; 30–60 Hz) and high-gamma (HG; 60–100 Hz) oscillations also occur while animals engage in theta-eliciting behaviors (Bragin et al., 1995; Burchell et al., 1998; Montgomery and Buzsáki, 2007; Belluscio et al., 2012; Schomburg et al., 2014). In CA1, LG is believed to originate from CA3 neurons synapsing onto CA1 pyramidal cells and interneurons and can be detected in stratum radiatum (Schomburg et al., 2014; Lasztóczi and Klausberger, 2014, 2016; Lopez-Pigozzi et al., 2016). On the other hand, HG would result from the temporoammonic pathway from entorhinal cortex to CA1 (Chrobak and Buzsáki, 1998) and is best observed in stratum lacunosum-moleculare (Scheffer-Teixeira et al., 2012; Schomburg et al., 2014). The subdivision of the traditional gamma range into subbands based on layer of occurrence and efferent region is thought to serve as means of information routing to CA1 (Colgin et al., 2009), which, in turn, would act as a comparator of different types of information (Lee et al., 2004).

The term “comodulation” generally denotes the interaction between specific features of two oscillations, such as instantaneous phase, frequency, and amplitude. When oscillations have different frequencies, the interaction is called cross-frequency coupling (CFC). Among other functions, CFC was proposed to underlie communication between brain areas, sensory demodulation, multi-item or sequential working memory, and phase precession (Jensen and Colgin, 2007; Axmacher et al., 2010; Canolty and Knight, 2010; Jirsa and Müller, 2013; Hyafil et al., 2015). In CA1, several results converge in showing that theta phase modulates the amplitude of LG and HG (Colgin et al., 2009; Scheffer-Teixeira et al., 2012; Schomburg et al., 2014; Lasztóczi and Klausberger, 2014, 2016; Lopez-Pigozzi et al., 2016). In addition, theta has been recently shown to modulate the amplitude of an even faster rhythm, which has been called high-frequency oscillations (HFOs; 120–160 Hz; Scheffer-Teixeira et al., 2012; Tort et al., 2013). However, although gamma oscillations have been widely reported, theta-associated HFOs remain relatively unknown. Nevertheless, given the putative importance of neuronal oscillations to physiologic and pathologic brain functioning (Bragin et al., 1999; Wang, 2010; Brankačk et al., 2012; Caixeta et al., 2013), it is likely that theta-associated HFOs have functional roles yet to be identified.

Possible reasons for the underreporting of theta-associated HFOs include (1) lack of proper signal analysis techniques that allow their identification (such as CFC tools); (2) lower importance attributed to the highest part of the local field potential (LFP) spectrum, which is often ignored (many consider oscillations faster than 100 Hz to represent extracellular spikes and not true oscillations; Scheffer-Teixeira et al., 2013); (3) the high prevalence of recordings from the pyramidal cell layer (to capture spiking activity), whereas theta–HFO coupling has been observed in more superficial layers (Tort et al., 2013); and (4) differences in recording techniques, such as electrode impedance, contact surface, and shape (single wires versus tetrodes vs probe contacts) potentially influencing HFO detection.

In the present work, we make a step toward better understanding theta-associated HFOs by outlining how these oscillations can be detected. To that end, we use spectral and CFC analyses to investigate different electrode types, recording locations, and behavioral states. We also contrast HFO activity within theta cycles with LG, HG, and multiunit activity. The results highlight unique properties of HFOs that separate them from gamma oscillations, as well as from spike contamination of LFP signals. This work should help in identifying theta-associated HFOs in other laboratories worldwide.

## Materials and Methods

### Subjects

We used seven male Wistar rats from our breeding colony (2–3 mo old; 300–400 g). Animals were maintained in a 12:12-h light-dark cycle, and recording sessions were performed during the light phase. All animal manipulations and care were approved by our local institutional ethics committee (CEUA/UFRN, protocol number 060/2011) in accordance with National Institutes of Health guidelines. These same animals were used in a recent publication of ours on cross-frequency phase-phase coupling (Scheffer-Teixeira and Tort, 2016).

### Surgery

Animals were anesthetized with a mixture of xylazine (10 mg/kg) and ketamine (100 mg/kg); supplementary doses of ketamine were injected if needed. After the skull was exposed and cleaned, target areas (fixation points and recording site) were marked using a stereotaxic apparatus and drilled. Six stainless steel screws were used for cap fixation. Two additional screws in the occipital bone (in contact with the dura mater) served as ground and reference for all electrodes. A thin layer of dental cement was applied around them. The silicon probe was lowered into the target area (CA1 region; AP, –3.6 mm; ML, 2.5 mm; DV, ∼2 mm, but variable across animals). Ongoing neural activity was monitored during probe placement and impedance measurement. While passing through the CA1 pyramidal layer, successive electrode contacts transiently exhibited low-frequency spikes. After insertion, the probe was secured to the skull with dental cement. The assessment of electrode location was performed by benchmark electrophysiological signatures during recordings sessions (Fig. 2). We used six custom-designed silicon probes manufactured by NeuroNexus (four probes with 4320-μm^2^ contact areas, one with 703-μm^2^ contact area, and one with 177-μm^2^ contact area). The probes had 16 channels in a linear vertical design with interelectrode distance of 100 μm. Contacts spanned from the parietal cortex above the hippocampus to CA1 layers; because of differences in final probe depth among animals, the span of recorded locations across animals is larger than a single probe span (Fig. 3*B*). In one animal, we implanted an independent movable microdrive consisting of three tetrodes composed of 12.5-μm-diameter platinum-iridium wires and four single 50-μm-diameter tungsten wires, which were lowered until the stratum alveus/oriens. After surgery, animals received analgesics and local and systemic antibiotics and were monitored for 1 wk.

### Recordings

After 1 wk of recovery, we recorded electrophysiological signals and videorecorded spontaneous behavior of the animals in an open field (1 × 1 m) in ∼4- to 5-h sessions. We used RHA 2116 (Intan Technologies) 16-channel recording system. The signals were amplified 200×, bandpass filtered between 1 Hz and 7.5 kHz, and digitized at 25 kHz. For LFP analysis, we filtered the raw data between 1 and 500 Hz and down-sampled to 1000 Hz. For multiunit activity, we filtered the raw data between 800 Hz and 8 kHz and obtained the timestamps of spikes using thresholds established by visual inspection.

### Histology

After the recording sessions, animals were killed for histologic validation of electrode positioning. To that end, we cut the brain in coronal sections and used Nissl staining to visualize probe/wire tracking in the parietal cortex and hippocampus.

### Behavioral classification

Epochs of active waking (aWK) and REM sleep were identified by inspection of electrophysiological signals and videorecordings. aWK was defined as periods of theta activity and visible movements, and REM sleep was defined by the presence of theta, absence of movements, sleep postures, and preceding slow-wave sleep.

### Data analysis

We used custom-written and built-in routines in Matlab (MathWorks). We also used routines from two third-party toolboxes: EEGLAB (Delorme and Makeig, 2004) and CircStat (Berens, 2009).

### Power spectral density and 1/f fitting

We computed power spectral densities (PSDs) using the *pwelch* function (Signal Processing Toolbox; 2-s Hamming windows with 1-s overlap). Time–frequency power decompositions were obtained with the *spectrogram* function (Signal Processing Toolbox) and used to assist the classification of sleep-wake states. For comparing power of individual fast oscillations (LG, HG, or HFO) between aWk and REM sleep (Fig. 5*C*), we first fitted a 1/*f* curve using PSD values around the frequency band of interest and then obtained a normalized peak power value by subtracting the 1/*f* fit from the actual peak power value (Scheffzük et al., 2013).

### Filtering, amplitude, and phase extraction

Filtering was achieved with the *eegfilt* function of the EEGLAB toolbox. The amplitude envelope and instantaneous phase of filtered LFP signals were obtained as the absolute value and angle of the analytic signal representation, respectively (*hilbert* function; Signal Processing Toolbox).

### Phase-amplitude coupling and comodulation maps

We estimated phase–amplitude coupling (PAC) strength using the modulation index (MI) described in detail elsewhere (Tort et al., 2008, 2010a; Scheffer-Teixeira et al., 2012; Caixeta et al., 2013). In brief, the MI measures how much a mean amplitude distribution over phase bins deviates from the uniform distribution. The comodulation map, or “comodulogram,” is obtained by expressing the MI computed for multiple frequency pairs (bandpassed LFP signals) by means of a 2D pseudocolor map, in which the *x*-axis denotes the phase frequency, and the *y*-axis the amplitude frequency (that is, a warm color in a given comodulogram entry means that the phase of the X frequency modulates the amplitude of the Y frequency). The comodulograms were constructed using 0–20 Hz (2-Hz steps, 4-Hz bandwidth) and 30–300 Hz (5-Hz steps, 10-Hz bandwidth) as phase and amplitude frequencies, respectively.

In the 2D pseudocolor maps of Fig. 3*C*, the *x*-axis denotes the amplitude-providing frequency (25–200 Hz, 5-Hz steps, 10-Hz bandwidth), the *y*-axis denotes anatomic depth (zero is pyramidal cell layer of the hippocampus), and color represents the normalized PAC strength between the theta phase and the amplitude of the fast oscillation in *x*-axis; the normalization is performed for each fast oscillation, obtained by normMI = [MI – min(MI)]/[max(MI) – min(MI)], where max(MI) and min(MI) denote the maximum and minimum MI values across depths. In Fig. 8A, the *x*-axis denotes the phase-providing frequency (0–16 Hz, 2-Hz steps, 4-Hz bandwidth), the *y*-axis denotes anatomic depth, and color represents PAC strength between the *x*-axis frequency and the amplitude of the fast oscillatory activity under study. The continuous time series of theta–HFO MI values shown in Fig. 5*A* was obtained using 30-s windows with 5-s overlap.

For the comodulograms and phase-amplitude coupling analyses, the amplitude and phase–time series were obtained from the same electrode. Thus, the local theta served as the phase reference. But we note that using either the local LFP or another (fixed) channel to extract the theta phase reference yield similar coupling strength for the fast local oscillation. This is because (1) theta is highly coherent along the parietal–CA1–dentate axis (Lubenov and Siapas, 2009), (2) the estimation of theta phase does not depend on theta amplitude (assuming a reasonable level of signal-to-noise ratio) and is therefore independent of the variability in theta amplitude along the dorsoventral axis, and (3) the modulation index does not take into account the preferred phase of maximal amplitude (i.e., the phase reversal of theta below the pyramidal cell layer does not influence coupling strength). The exception was Fig. 6, which displays the preferred theta phase of maximal amplitude for the different fast oscillations. In this case, for all fast oscillations, the reference theta phase was fixed and taken from stratum oriens-alveus (note that the instantaneous theta phase is the same from the pyramidal cell layer up to the parietal cortex; Fig. 2 and Lubenov and Siapas, 2009).

### Surrogates

In addition to comparing MI levels between aWK and REM sleep states, we also used surrogate analysis to infer the very existence of PAC (Tort et al., 2010a). To that end, we obtained surrogate MI values using a “split-invert-splice” method: while the phase time series was left intact, we randomly split the amplitude time series and concatenated the two parts inverted. For each comodulogram in Fig. 3*A*, we computed a distribution of 200 surrogate comodulograms; actual MI values below at least one surrogate MI value were set to zero.

### Phase–energy plots

In Fig. 6, we estimated the preferred theta phase (i.e., the phase of maximal amplitude) of each fast oscillation of interest by visual inspection of phase–energy plots (see Fig. 6*A* for examples). To obtain the phase–energy plot, we used Morlet wavelet spectral decomposition using scaled pseudofrequencies of 20–200 Hz in 2-Hz steps. We then calculated the mean wavelet amplitude over theta phase bins of 2°. To compensate for the 1/*f* decay, the mean amplitude of each frequency was normalized by the average across all phase bins. In this analysis, a fixed theta reference at stratum oriens-alveus was used (which provides the same phase reference as in stratum pyramidale but with a better signal-to-noise ratio).

### Independent component analysis

To obtain a better estimate for the spatial origin of the fast oscillations recorded with the linear probes, we used independent component analysis (ICA) as described in Schomburg et al. (2014). To that end, we first filtered the LFPs >30 Hz and then used the KDICA algorithm (Chen, 2006); aWK and REM states were analyzed separately. The resulting independent components (ICs) were used as the amplitude-providing signal for the comodulogram analysis, and a stratum oriens-alveus LFP provided the phase signal. The comodulograms were next inspected and used to classify the ICs as containing LG, HG, or HFO. The absolute value of the voltage loadings of ICs exhibiting isolated gamma or HFO activity were interpreted as an estimate of the spatial source (see Fig. 4 for examples).

### Fast oscillatory bursts per theta cycle

For each fast frequency (LG, HG, and HFO), we defined the occurrence of an oscillatory burst as the crossing of the amplitude envelope of a threshold of 4 SD above the mean amplitude. We identified individual theta cycles by the timestamps of consecutive valleys (i.e., a cycle was defined to start/finish at the valley) in bandpass-filtered LFPs from 4 to 20 Hz, a frequency range previously shown to provide a good fit to asymmetric theta cycles (Belluscio et al., 2012). We then quantified the fast oscillatory bursts per theta cycle. In this analysis, we used a channel in stratum oriens-alveus as theta reference for all fast oscillations.

### PAC strength controlled for theta amplitude

In Fig. 5*E*, we compare PAC strength between theta phase and the amplitude of fast oscillations (LG, HG, and HFO) as a function of theta amplitude. This was achieved by first obtaining the phase and amplitude time series for the entire signal. We then *z*-scored the mean theta amplitude of each theta cycle. Finally, we classified theta cycles by their mean amplitude (in units of *z* score) and computed PAC strength using only time series periods associated to theta cycles of a fixed theta amplitude bin (bin width = 0.2 *z* score). We excluded bins containing <10 s of signal to ensure reliable MI estimation.

### Spike-phase coupling

We estimated spike-phase coupling (SPC) strength from the probability distribution of spikes over phase bins (extracted from bandpass-filtered LFPs). We fitted a von Mises distribution and used the concentration parameter (kappa) as a measure of SPC (Berens, 2009). The higher the kappa, the higher the SPC.

### Spike- and oscillation-triggered LFP averages and time–energy plots

To construct triggered LFP averages, we used 500-ms windows around the trigger timestamp (ripples and theta peaks in Fig. 2, spike times in Fig. 9). We averaged either raw LFP signals (Fig. 2*B* left panel and Fig. 9) or filtered signals (Fig. 2*B*, right, and 2*C*). The time–energy plots in Fig. 9 were obtained from the spike-triggered averages of the amplitude envelope of filtered LFP signals (20–250 Hz, 1-Hz steps, 10-Hz bandwidth). In these plots, the amplitude of each frequency was normalized by its maximum value to control for 1/*f*.

### Statistics

**Figure 1.**
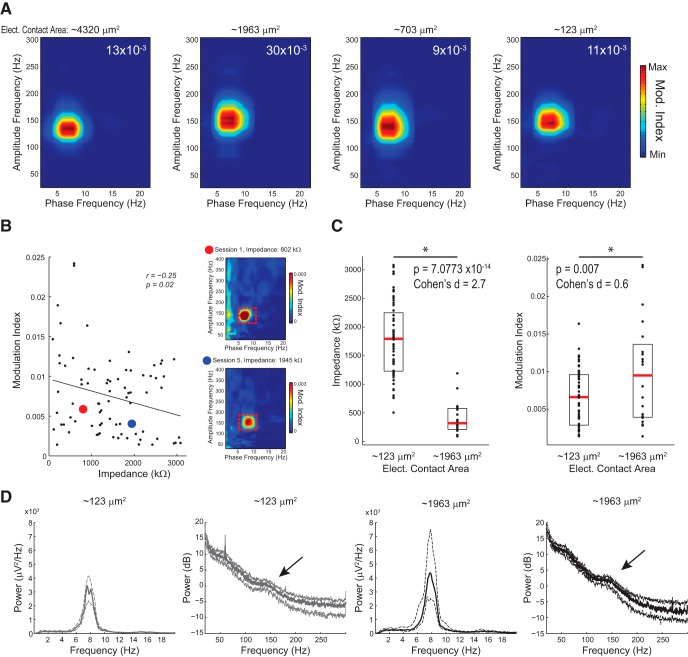
Electrode impedance does not considerably influence comodulation assessment. ***A***, Phase-amplitude comodulograms computed for parietal cortex LFPs recorded during REM sleep from four electrode types differing in contact area. Top right numbers denote maximum modulation index (Mod. Index). Electrode type and material, from left to right: linear planar silicon probe, tungsten single wire, linear planar silicon probe, platinum-iridium tetrode wire. Each comodulogram was obtained from one recording session in one animal (three different animals were used; the single and tetrode wire recordings were from the same animal and session). ***B***, Left, scatter plot of electrode impedance and theta–HFO modulation index. Data obtained from 16 electrodes positioned in the parietal cortex in the same animal across five sessions. The thick line depicts the linear fit. Right, example comodulograms obtained from the same electrode in sessions 1 and 5 (red and blue circles in the scatter plot; electrode area: 123 μm^2^); red dashed box indicates region used to compute the strength of theta–HFO comodulation. Note a substantial increase in impedance along with a mild decrease in comodulation. ***C***, Electrode impedance (left) and theta–HFO modulation index (right) measured for electrodes of 123 μm^2^ (*n* = 12) and 1963 μm^2^ (*n* = 4) contact area across five sessions for the same animal as in ***B***. Boxes indicate 25th and 75th percentiles, red trace denotes the median, and dots show individual values. Note that although larger electrodes have much lower impedance, comodulation strength does not differ as much. ***D***, Mean power spectra for the same recordings as in ***B*** and ***C***. Thick and dashed lines represent median and 25th–75th percentiles, respectively. Arrows highlight a power peak in the HFO range in both electrode types.

We opted to exclude *p*-value–only based decisions because of recent concerns in the field and as an effort to increase statistical quality (Wasserstein and Lazar, 2016). Therefore, in addition to *p*-values (calculated using two-tailed tests), we based our statistical inferences on 95% confidence intervals (CIs) of the mean, effect size (Cohen’s *d*; calculated as suggested by Lakens [2013]), and more transparent data presentation (Weissgerber et al., 2015). We avoided nested data by analyzing one measure per animal, and unless noted otherwise (e.g., in Fig. 1), the sample size is the number of animals and not electrodes, which could inflate power. Each sample consisted of either the mean or the median of scores (e.g., MI, power, or phase) over all eligible electrodes per animal. Statistical analyses are summarized in Table 1.

**Table 1.** Statistical table

Results	Data structure	Type of test	Sample size	CI/*p*-value
Fig. 1*B*: correlation between CFC strength and impedance	Assumed normal distribution of the errors	Pearson correlation tested using *t* test	*n* = 80 electrodes of 1 animal over 5 sessions	ρ = –0.255 *p* = 0.0224
Fig. 1*C*: impedance values for two different sizes of electrodes	Assumed normal distribution	Independent *t* test; *t*-based confidence intervals for the mean	*n* = 20 single wires and 60 tetrode wires electrodes of 1 animal over 5 sessions	CI_95_ = [1072;1672] Cohen’s *d* = 2.7 *p* = 7.0773 × 10^−14^
Fig. 1*C*: CFC strength for two different sizes of electrodes	Assumed normal distribution	Independent *t* test; *t*-based confidence intervals for the mean	*n* = 20 single wires and 60 tetrode wires electrodes of 1 animal over 5 sessions	CI_95_ = [0.9; 5.8] × 10^−3^ Cohen’s *d* = 0.6 *p* = 0.007
Fig. 3*A*: comodulograms were compared to a surrogate distribution obtained by randomly splitting and inverting the amplitude time series	Assumed nonnormal distribution	Surrogate test	200 surrogates for each channel	Each *x–y* entry was set to zero (dark blue color) if observed *p* > 0; otherwise, the original value was kept.
Fig. 5*C*: LG power comparison for aWK and REM sleep	Assumed normal distribution	Dependent *t* test; *t*-based confidence intervals for the mean	*n* = 7 animals (mean across electrodes)	CI_95_ = [–1.3; 2.73]; Cohen’s *d* = 0.33; *p* = 0.42
Fig. 5*C*: HG power comparison for aWK and REM sleep	Assumed normal distribution	Dependent *t* test; *t*-based confidence intervals for the mean	*n* = 7 animals (mean across electrodes)	CI_95_ = [–0.39; 1.1]; Cohen’s *d* = 0.44; *p* = 0.29
Fig. 5*C*: HFO power comparison for aWK and REM sleep.	Assumed normal distribution	Dependent *t* test; *t*-based confidence intervals for the mean	*n* = 7 animals (mean across electrodes)	CI_95_ = [0.15; 0.58]; Cohen’s d = 1.57; *p* = 0.006
Fig. 5*C*: Theta–LG coupling strength comparison for aWK and REM sleep	Assumed normal distribution	Dependent *t* test; *t*-based confidence intervals for the mean	*n* = 7 animals (mean across electrodes)	CI_95_ = [–0.22; 0.11] × 10^−3^; Cohen’s *d* = 0.31; *p* = 0.45
Fig. 5*C*: Theta–HG coupling strength comparison for aWK and REM sleep	Assumed normal distribution	Dependent *t* test; *t*-based confidence intervals for the mean	*n* = 7 animals (mean across electrodes)	CI_95_ = [0.03; 2.14] × 10^−3^; Cohen’s *d* = 0.95 *p* = 0.045
Fig. 5*C*: Theta–HFO coupling strength comparison for aWK and REM sleep	Assumed normal distribution	Dependent *t* test; *t*-based confidence intervals for the mean	*n* = 7 animals (mean across electrodes)	CI_95_ = [3; 10.8] × 10^−3^; Cohen’s *d* = 1.64; *p* = 0.005
Fig. 5*D*: Theta power comparison for aWK and REM sleep	Assumed normal distribution	Dependent *t* test; *t*-based confidence intervals for the mean	*n* = 7 animals (mean across electrodes)	CI_95_ = [0.11; 0.2]; Cohen’s *d* = 3.19; *p* = 1.52 × 10^−4^

## Results

### Theta-associated HFOs can be detected with different electrode types

We first sought to investigate whether the underreporting of HFOs could be due to differences in electrode types among laboratories. We have previously obtained prominent records of theta-associated HFOs using low-impedance, single tungsten wires of 50-μm diameter (Scheffer-Teixeira et al., 2012). We were interested in knowing whether HFOs can also be detected with electrodes of different geometry, such as planar probe contacts and the thinner wires used in tetrodes. We thus compared LFP recordings obtained from single 50-μm-diameter tungsten wires (1963 μm^2^ contact area), from multisite probes of large (4320 μm^2^) and intermediate (703 μm^2^) contact areas and from tetrodes made of 12.5-μm-diameter platinum-iridium wires (123 μm^2^ contact area).

We could detect prominent theta–HFO coupling in the parietal cortex during REM sleep in all electrode types (Fig. 1*A*). The highest modulation index was observed for an LFP recorded with the single 50-μm-diameter tungsten wire (Fig. 1*A*, middle left). We next investigated whether electrode impedance affects HFO detection. We found that electrode impedance (estimated *in vivo*) has only mild impact on theta–HFO coupling strength (Fig. 1*B*, *C*). The scatter plot between CFC strength and impedance shown in Fig. 1*B* reveals a weak negative correlation (Pearson’s *r*: –0.255, *p* = 0.022; all electrodes in a same animal along five sessions). The red and blue dots in Fig. 1*B* highlight changes in impedance and CFC strength in the same electrode between the first and last sessions. Despite the major increase in impedance (2.4-fold, from 802 to 1945 kΩ), the last recording session exhibited theta–HFO coupling as prominent as in the first session. Moreover, although impedance was significantly lower in single 50-μm-diameter wires than in 12.5-μm-diameter tetrode wires (Fig. 1*C*, left; *p* = 7.077 × 10^−14^, *t* test), these electrodes exhibited comparable levels of theta–HFO coupling (Fig. 1*C*, right). Only 4 of 20 LFP samples (20%) recorded with the thicker wires had higher theta–HFO coupling than all samples (*n* = 60) collected with tetrode wires. The median level of theta–HFO coupling was mildly higher for 50- than 12.5-μm-diameter wires. Although this difference reached statistical significance (Fig. 1*C*, right; *p* = 0.007, *t* test), the effect size measured using Cohen’s *d* was 0.6 (associated with 76.4% overlap), much smaller than the observed effect size for the difference in impedance, 2.7 (associated with 17.7% overlap). Finally, we computed PSDs and found that both 50- and 12.5-μm-diameter wires had comparable levels of theta power; moreover, both electrode types exhibited a clear power bump in the HFO range (120–160 Hz; Fig. 1*D*). In all, we conclude that differences in electrode geometry and impedance have only minor influences on HFO detection.

### Recording location and behavioral state are the main factors determining HFO detection

**Figure 2.**
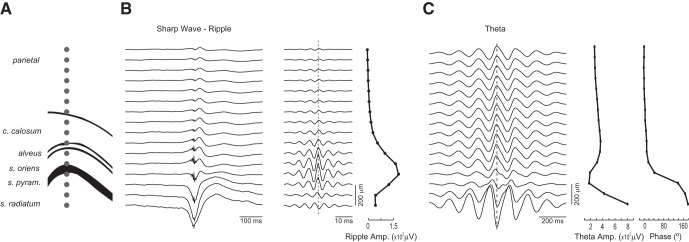
Estimation of electrode location in linear probes. ***A–C***, Electrode placement across the dorsoventral axis was estimated (***A***) using the amplitude of sharp-wave ripples (***B***), the amplitude and phase of theta oscillations (***C***), and presence of spikes (not shown).

**Figure 3.**
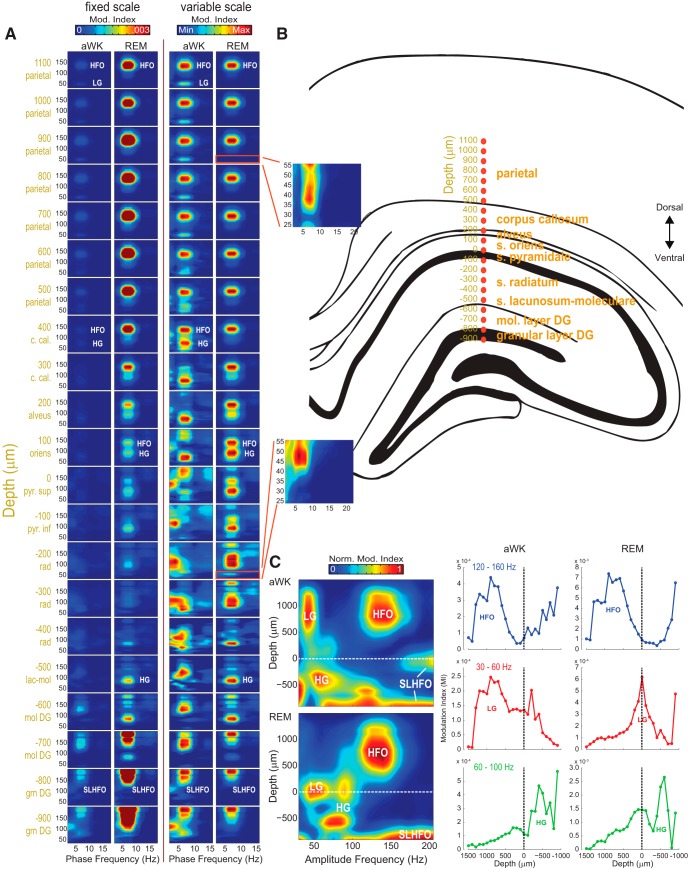
Comodulation patterns are layer and state dependent. ***A***, Mean comodulograms (*n* = 4 rats) during aWK and REM sleep for recording sites linearly spaced from parietal cortex to hippocampus. Modulation indexes are plotted using both fixed (left) and variable (right) pseudocolor scales to allow quantitative and qualitative comparisons, respectively. Insets are rescaled and zoomed-in views of the LG range. ***B***, Estimated electrode locations (adapted from Paxinos and Watson, 2004). Depth units are relative to the hippocampal pyramidal layer. ***C***, Left, 2D maps of normalized theta coupling strength as a function of amplitude frequency and anatomic depth (average over four rats). For each amplitude frequency, 0 denotes the minimum modulation index and 1 the maximum. Right, mean theta coupling strength (*n* = 4 rats) plotted as a function of recording depth separately for each fast oscillation (LG, HG, and HFO) and brain state (aWK and REM). LG, 30–60 Hz; HG, 60–100 Hz; HFO, 120–160 Hz.

In our previous work, we showed that PAC patterns depend on recording depth across the CA1–dentate axis, with theta–HFO coupling being most apparent in more superficial layers than theta–HG coupling (Scheffer-Teixeira et al., 2012). Here, we extend these results by mapping comodulation patterns from multiple, linearly spaced contacts from the parietal cortex to the hippocampus, separating the analyses in two behavioral states: aWK and REM sleep (see Materials and Methods). Fig. 2 shows a representative example of the estimation of contact positions in a multisite linear probe using benchmark electrophysiological signatures, such as sharp-wave reversal, ripple power, theta reversal, and theta power (Bragin et al., 1995; Ylinen et al., 1995). We classified recording depth using as reference the distance from the CA1 pyramidal cell layer (depth = 0; positive and negative depths denote regions above [dorsal] and below [ventral] s. pyramidale, respectively; Fig. 3*B*). Fig. 3*A* shows phase-amplitude comodulograms computed at the different recording depths; the left column shows all comodulograms at the same MI scale, and the right column displays each comodulogram on its own MI scale to allow qualitative comparisons of coupling patterns. The comodulograms revealed the same qualitative pattern in each animal and corroborated our previous observations (Scheffer-Teixeira et al., 2012): theta–HFO coupling was detected above the pyramidal cell layer, whereas theta–HG coupling presented a richer picture: it was high in the stratum oriens and pyramidal cell layer, decreased in the stratum radiatum, and achieved maximum strength in the stratum lacunosum-moleculare (Fig. 3*A*). In both aWK and REM sleep, theta–HFO coupling was most apparent at recordings from the parietal cortex. Interestingly, a careful inspection of Fig. 3*A* reveals that the transition from theta–HFO to theta–HG coupling occurs at the corpus callosum during aWK (comodulograms at 300 and 400 μm); however, during REM sleep, the transition between coupling patterns occurs much deeper, around the CA1 pyramidal cell layer (comodulograms at 100, 0, and –100 μm). These results therefore show that recording location across the parietal–hippocampal axis is a crucial factor for the detection of theta–HFO coupling.

During aWK, we observed weak levels of theta–LG coupling in the parietal cortex (co-occurring with theta–HFO coupling) and in stratum radiatum (Fig. 3*A*). During REM sleep, theta–LG coupling was not as apparent in the comodulograms, except for a small comodulation “island” in the LG range in stratum radiatum (in –200 μm from the pyramidal cell layer; Fig. 3*A*). Finally, we also observed an unexpected coupling between delta phase and the amplitude of oscillations ∼100 Hz in some electrode depths during aWK (Fig. 3*A*, variable scale); this pattern may correspond to respiratory inputs (Yanovsky et al., 2014; Nguyen Chi et al., 2016; Lockmann et al., 2016), whose coupling to gamma disappears during REM sleep (Zhong et al., 2017) and will be addressed in an upcoming study.

**Figure 4.**
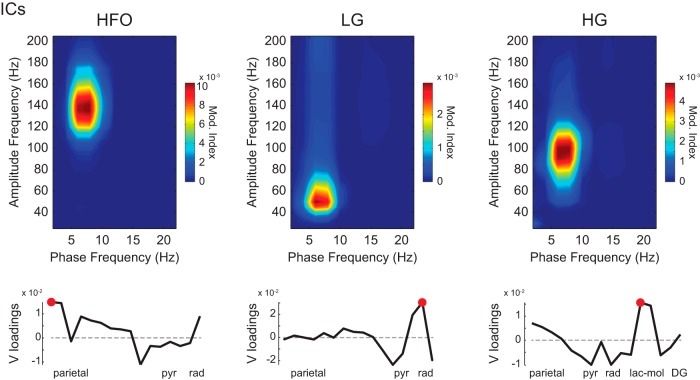
ICA decomposition of LFPs isolates phase-amplitude coupling patterns and suggests spatial sources. Top, representative phase-amplitude comodulograms computed for ICs that isolate activity of specific fast oscillations, as labeled. Each IC is a weighted sum of LFPs recorded with a linear probe across the dorsoventral axis (see Schomburg et al., 2014). Bottom, voltage (V) loadings as a function of recording depth. Red dots mark highest absolute V loadings.

To further compare coupling patterns across regions, in Fig. 3*C* we plot absolute MI values as a function of recording depth for each fast oscillation separately (right), as well as 2D maps of normalized theta coupling strength as a function of the amplitude frequency and depth (left; the normalization was performed for each amplitude frequency; see Materials and Methods). Consistent with Fig. 3*A*, these plots reveal highest theta–HFO coupling strength at the parietal cortex and highest theta–HG coupling near the hippocampal fissure. Theta–LG coupling was weak but apparent at the parietal cortex and stratum radiatum during aWK. Curiously, theta–LG coupling seemed to appear near the pyramidal cell layer during REM sleep. Although at first glance such a finding is very interesting, we suspect it reflects a “contamination” of the LG range by a “tail” (or leakage) of theta–HG coupling, which considerably increases during REM sleep. We also note that a similar phenomenon occurs for the estimation of theta–HFO coupling in the dentate gyrus, which gets heavily contaminated by theta modulation of spike-leaked HFOs (SLHFOs; discussed below). Finally, using the same linear probe data, we also computed phase-amplitude comodulograms for ICs of fast oscillatory activity as introduced in Schomburg et al. (2014). The voltage loadings tended to be highest at the parietal cortex for ICs selectively exhibiting theta–HFO coupling, at stratum radiatum for ICs with selective theta–LG coupling, and at stratum lacunosum-moleculare for ICs exhibiting theta–HG coupling (see Fig. 4 for examples).

**Figure 5.**
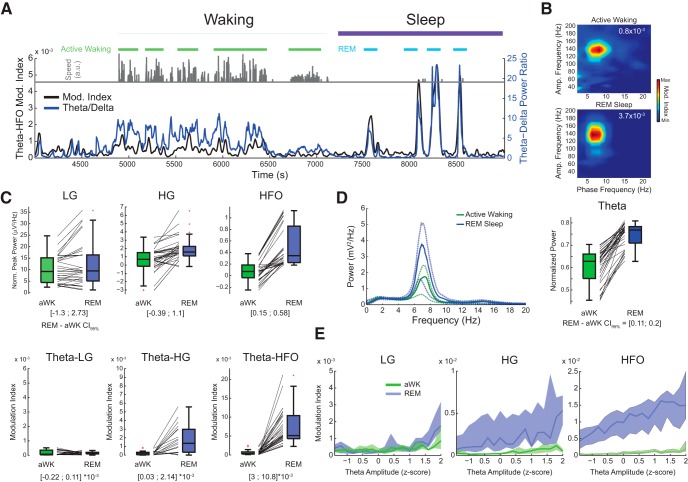
Phase-amplitude coupling is most prominent during REM sleep. ***A***, Example of a continuous recording including epochs of waking and sleep. Periods of aWK and REM sleep are indicated by light green and blue horizontal traces. Gray bars on top show instantaneous animal speed; theta/delta power ratio (blue) and theta–HFO modulation index (black) are shown in the bottom plot. ***B***, Comodulograms for aWK and REM epochs shown in ***A***. ***C***, Top, normalized peak power for LG (30–60 Hz), HG (60–100 Hz), and HFO (120–160 Hz) during aWK and REM. The normalization was obtained by subtracting the 1/*f* fit (see Materials and Methods and Scheffzük et al., 2011). Each data point corresponds to an electrode (LG, *n* = 30 electrodes across 7 rats; HG, *n* = 20; HFO, *n* = 36). CI_95%_ for the paired difference between states was computed using the mean value over eligible electrodes for each rat (*n* = 7 animals; for Cohen’s *d* and *p*-values, see Table 1). Bottom, modulation index computed for theta phase and the amplitude of each frequency range. Notice larger theta–HG and theta–HFO coupling during REM sleep. ***D***, Power spectral density (left) and mean theta power (right) during aWK and REM. Continuous thick lines represent median, and dashed thin lines 25th–75th percentiles. ***E***, Phase-amplitude coupling strength as a function of theta amplitude for different fast oscillations during aWK and REM sleep. In these analyses, eligible electrodes were selected based on inspection of comodulograms; for LG, we used recordings from s. radiatum, s. pyramidale, and parietal cortex; for HG, from s. lacunosum-moleculare and s. pyramidale; for HFO, from stratum oriens-alveus and parietal cortex.

The left column of Fig. 3*A* shows that the maximum coupling strength substantially increases from aWK to REM sleep for theta–HG and theta–HFO coupling, but not for theta–LG coupling (note also *y*-axis scales in Fig. 3*C*). We proceeded to further investigate quantitative differences between these two theta states. Fig. 5*A* shows a representative example of variations of theta power (expressed as theta/delta ratio) and theta–HFO coupling strength in time. Note the high similarity of the two time-series across sleep-awake states and the highest values during REM sleep (see also Fig. 5*B*). We next compared the power of the different fast oscillations between the two behavioral states. The PSD of aWK periods displayed higher power levels for frequencies >50 Hz than the PSD of REM epochs; however, the aWk PSD did not exhibit a clear power bump as reported in Fig. 1*D* (not shown, but see Scheffzük et al., 2011, 2013). To avoid spurious estimates resulting from generalized augmented power (probably due to muscular activity during aWk), we normalized power by subtracting, from the original PSD, the 1/*f* fit computed using power values around the frequency band of interest (Scheffzük et al., 2013; see Materials and Methods; the boxplot distributions show all electrodes across animals, but for computing CIs and *p*-values, each sample consisted of the mean over electrodes per animal). Under this framework, neither LG power nor HG power differed between aWK and REM sleep (Fig. 5*C*, top; REM-aWK CI_95_ = [–1.3; 2.73], *p* = 0.42, and [–0.39; 1.1], *p* = 0.29 for LG and HG power differences, respectively). On the other hand, HFO power significantly increased during REM sleep (REM-aWK CI_95_ = [0.15; 0.58], *p* = 0.006). At the group level, theta–LG coupling was not different between the two states (REM-aWK CI_95_ = [–0.22; 0.11] × 10^−3^, *p* = 0.45) but significantly increased for theta–HG (REM-aWK CI_95_ = [0.03; 2.14] × 10^−3^, *p* = 0.045) and theta–HFO coupling (REM-aWK CI_95_ = [3; 10.8] × 10^−3^, *p* = 0.005).

Theta power during REM sleep was also significantly higher than during aWK (Fig. 5*D*; REM-aWK CI_95_ = [0.11; 0.2], *p* =1.52 × 10^−4^). Differences in theta power between aWK and REM could thus explain the higher phase-amplitude coupling observed during REM. In Fig. 5*E*, however, we show that when controlling for theta amplitude, coupling strength was visibly higher during REM sleep (blue) compared to aWK epochs (green) for both HG and HFO, but not for LG, irrespective of power level. Therefore, our results show that comodulation strength highly depends on behavioral state and that the increase in theta–HG and theta–HFO coupling during REM sleep is not explained by changes in theta power.

### Different fast oscillations occupy different theta phases and theta cycles

**Figure 6.**
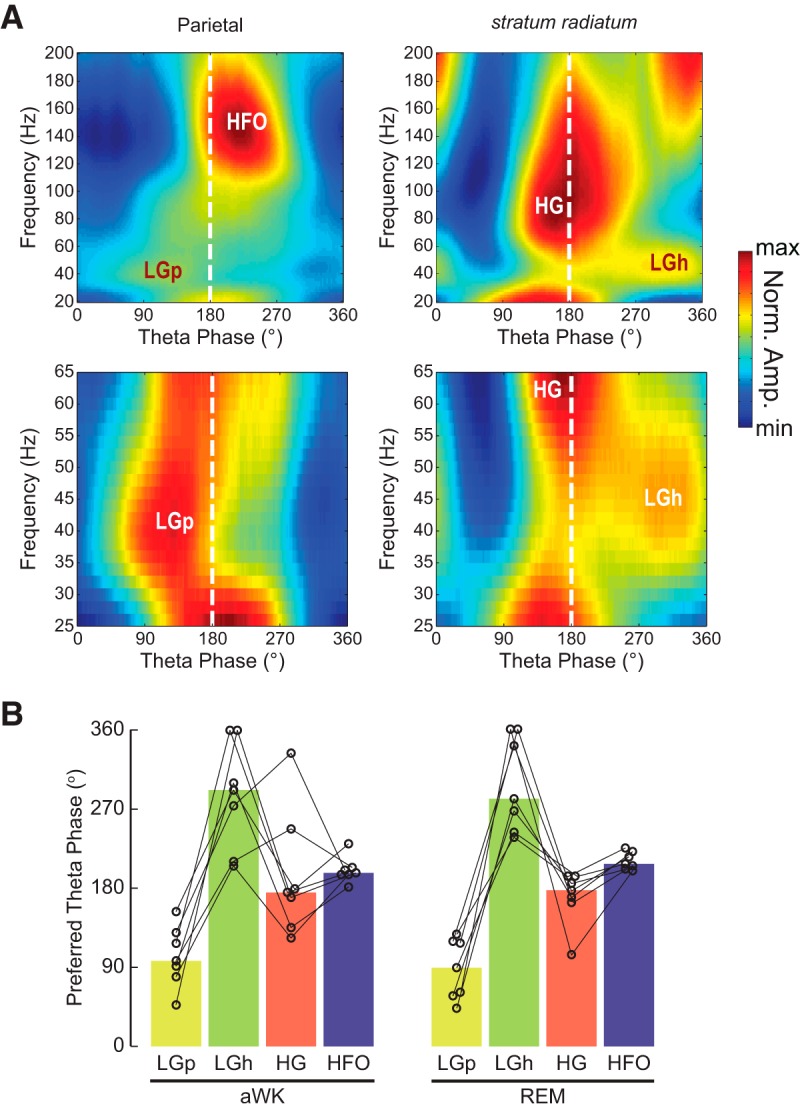
Preferred theta phase of maximal amplitude for different fast oscillations. ***A***, Top, representative phase–energy plots for LFPs recorded in the parietal cortex (left) and stratum radiatum (right) during REM sleep. Bottom, the same in rescaled and zoomed-in view to allow inspection of the LG range. Note different preferred theta phases for LG in parietal cortex (LGp) and hippocampus (LGh). ***B***, Preferred theta phase of maximal amplitude. Linked circles show data from the same animal. Bars depict the circular median over animals. For each fast oscillation and animal, we considered the electrode that displayed the maximum modulated activity at the corresponding frequency band on visual inspection of phase–energy plots (e.g., HFO was taken from the parietal cortex and HG from stratum lacunosum-moleculare). For all fast oscillations, we used the theta phase from an LFP at stratum oriens-alveus.

**Figure 7.**
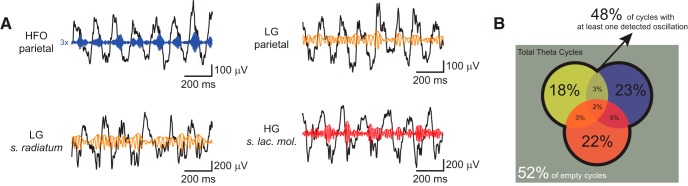
Different frequency bands use their own theta cycle. ***A***, Examples of raw traces along with filtered LFPs, as labeled. ***B***, Venn diagram of theta cycles classified according to the detection of oscillatory bursts. A burst event was defined to occur when the instantaneous amplitude envelope of the band-filtered signal crossed >4 SD from the mean. Percentages of theta cycles with oscillatory bursts include intersections. When discounting intersections, burst percentages sum to 48%. LG, yellow; HG, red; HFO, purple. Results are shown only for REM sleep epochs; results for aWK were similar. For each animal and fast oscillation, we analyzed one electrode, selected based on visual inspection of comodulograms. The electrode was located in the parietal cortex for HFO, at s. radiatum for LG, and at s. lacunosum-moleculare for HG. Percentages represent the mean over animals.

The results thus far show that the modulation of LG, HG, and HFO amplitude by theta phase is spatially segregated. We next investigated whether these fast oscillations temporally segregate in respect to theta cycle and theta phase (Figs. 6 and 7). During both aWK and REM sleep, we found amplitude peaks for different fast oscillations in different theta phases (Fig. 6*A*, *B*). Using theta phase above the pyramidal cell layer as reference (theta peak corresponds to 180°), we found that HG amplitude (recorded at s. lacunosum-moleculare) was maximal near the theta peak, and HFO amplitude (recorded in parietal cortex) was maximal right after the theta peak and HG maximal amplitude. Interestingly, we found that LG activity in parietal cortex (LGp) was maximal at the ascending theta phase, whereas LG in the hippocampus (LGh, recorded at s. radiatum) was maximal at the descending theta phase.

We also found evidence for segregation of the theta cycles in which different oscillatory bursts occur (Fig. 7). For each fast oscillation, we obtained the timestamps in which the amplitude envelope was 4 SD above its mean and classified theta cycles according to oscillatory bursts. Possible cases are theta cycles containing no peak of fast oscillation amplitude, an amplitude peak for just one fast oscillation, or amplitude peaks of two or three fast oscillations. We found that about half of all theta cycles did not contain any amplitude peak (52 ± 3% [mean ± SD across animals]). Among all theta cycles, we found that 18 ± 1% contained LG (10% exclusively), 22 ± 2% HG (12% exclusively), and 23 ± 2% HFO (13% exclusively); that is, 73% of theta cycles with a fast oscillatory burst were specific for LG, HG, or HFO (Fig. 7*B*). A minority of theta cycles contained two or more fast oscillatory bursts of different frequencies. In all, these results show that theta phase modulation of distinct fast oscillations tends to segregate into different theta cycles.

### Theta-associated HFOs differ from spike contamination of LFP signals

Previous reports have raised the possibility that fast LFP frequencies (>100 Hz) stem from spectral leakage of spike waveforms recorded extracellularly (Ray et al., 2008a, 2008b, 2008c; Ray and Maunsell, 2011; Belluscio et al., 2012). Namely, abrupt, nonsinusoidal deflections of the extracellular voltage lead to spurious fast oscillations in filtered signals (Kramer et al., 2008). We previously corroborated that such effects of extracellular spikes do occur and called the spurious LFP activity originated from it “spike-leaked HFOs” (SLHFOs; Scheffer-Teixeira et al., 2013; Tort et al., 2013). But we have also shown that not all LFP activity >100 Hz is due to spike contamination; in fact, we showed that theta-associated HFOs are genuine oscillations (Scheffer-Teixeira et al., 2013). Here we aimed to reproduce and extend these observations.

Spike contamination of the LFP signal in some cases can leak down to 100 Hz (Ray et al., 2008a). Accordingly, in our previous study, we showed that the frequency range of theta-coupled SLHFOs, as assessed by phase-amplitude comodulograms, may also comprise the HFO band (Scheffer-Teixeira et al., 2013). To separate SLHFO activity from genuine HFOs, in this work we filtered LFPs >150 Hz. Notice therefore that although we cannot avoid contamination of the estimation of theta–HFO coupling by theta–SLHFO coupling (as happens in the dentate gyrus; Fig. 3*C*), by filtering >150 Hz we can minimize contamination in the other direction, that is, contamination of the estimation of theta–SLHFO coupling by theta–HFO coupling.

**Figure 8.**
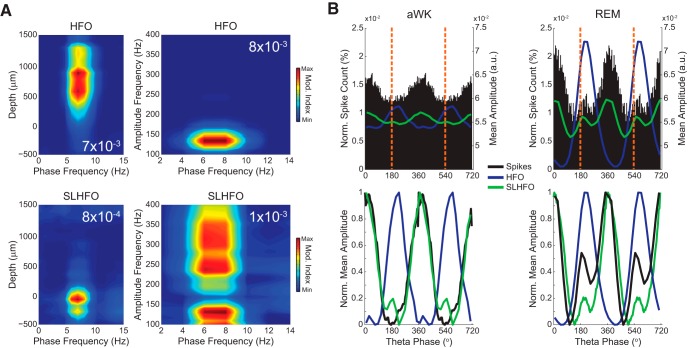
Spikes generate an artifactual fast LFP oscillation coupled to theta phase. ***A***, Left, mean depth comodulograms (*n* = 4 rats) for HFO (top) and SLHFO (>150 Hz; bottom). Theta–HFO coupling is strongest above the pyramidal cell layer, which is dominated by theta–SLHFO coupling. Right, mean comodulograms (*n* = 4 rats) for electrodes located in the parietal cortex (top) and the hippocampal pyramidal cell layer (bottom). Note genuine HFO and spurious SLHFO coupling to theta phase. ***B***, Top, spike probability (black bars; left *y*-scale) plotted along with the amplitude (right *y*-scale) of HFO (120–160 Hz; purple lines, parietal cortex recordings) and SLHFO (>150 Hz; green lines, pyramidal cell layer recordings) per theta phase during aWK and REM (group data; *n* = 4 rats). Dashed vertical orange lines indicate 180° and 540° (pyramidal theta peaks). Bottom, same as above, but with values normalized between 0 and 1. Note similar phase distributions between SLHFO amplitude and spike probability, which have a maximum near the trough of the theta wave.

Theta-associated HFOs are best detected above the pyramidal cell layer (Fig. 8*A*, top left) and appear in the phase-amplitude comodulogram as a circumscribed “island” between ∼110 and 160 Hz in the amplitude frequency axis (Fig. 8*A*, top right). On the other hand, SLHFO modulation by theta is most prominent at the pyramidal cell layer (Fig. 8*A*, bottom left) and in the dentate gyrus (see depths below –500 μm in Fig. 3*A*; see also Fig. 5 in Scheffer-Teixeira et al., 2012), and the phase-amplitude comodulogram displays a wide range of modulated amplitude frequencies (Fig. 8*A*, bottom right). Importantly, whereas HFO amplitude peaks after the theta peak (s. pyramidale reference), both SLHFO amplitude and CA1 multiunit activity are maximal near the theta trough (Fig. 8*B*). Interestingly, during REM sleep, the distribution of CA1 spikes over the theta cycle exhibits a second maximum near the theta peak (Poe et al., 2000; Senior et al., 2008), and SLHFO amplitude displays a similar bimodal profile (Fig. 8*B*, right). Of note, the phase of the second spiking activity peak matches the phase of maximal HFO amplitude within a theta cycle. It is therefore possible that the increase in HFO activity observed during REM sleep (Fig. 5) drives spiking activity in CA1 and leads this second peak in spike probability.

**Figure 9.**
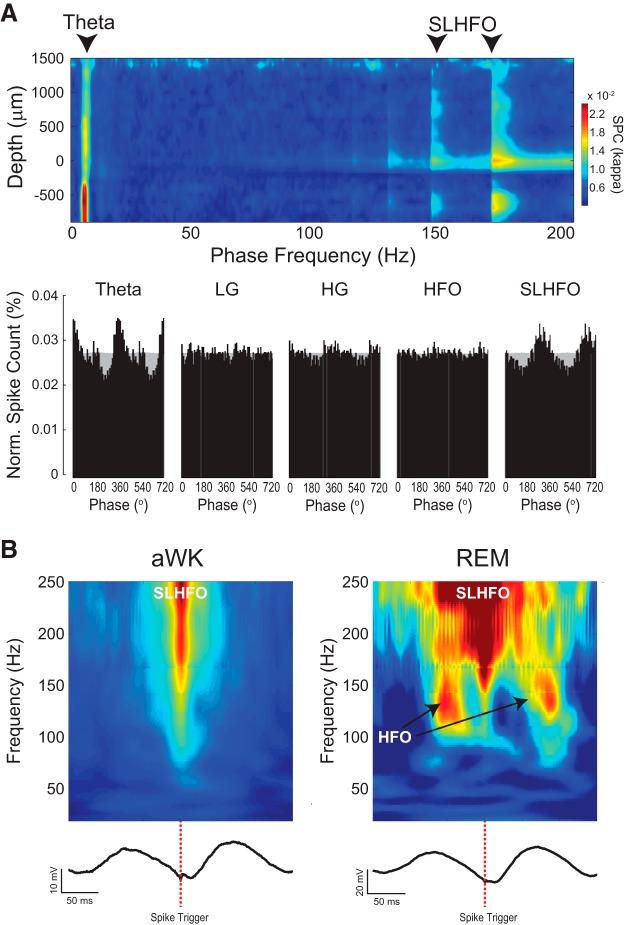
Spurious spike modulation by SLHFO. ***A***, Top, SPC strength (see Materials and Methods) as a function of frequency band and depth along the dorsoventral axis (pooled data across all REM sleep episodes in four animals). Note strong modulation by both theta and SLHFO. Bottom, spike-phase distribution for multiple frequency bands (black bars; oscillations were recorded from the pyramidal cell layer [depth = 0 μm]). Gray light bars show the phase probability, which is roughly uniform for each frequency. ***B***, Spectral decomposition of spike-triggered LFP traces can exhibit both SLHFO and HFO. Top, time–frequency LFP energy triggered by spike times during aWK and REM (average over four animals; electrodes located at depth 600 μm). Note the presence of SLHFO in both states, whereas HFO appears only during REM (arrows). Bottom, spike-triggered LFP average. Red dashed lines indicate spike time for referencing in both plots.

Multiunit activity couples to theta phase in both aWK and REM sleep (Fig. 8*A*). We next investigated the dependence of spike coupling on LFP frequency and depth across the parietal–hippocampal axis. In Fig. 9*A* (bottom), we show examples of spike–phase distributions of CA1 multiunit activity for multiple LFP oscillations recorded from the pyramidal cell layer during REM sleep. We observed prominent spike–phase coupling only for theta and SLHFO, but not for LG, HG, or HFO (these results refer to the multiunit spike pool and do not imply lack of coupling for single, well-isolated units). It should be noted that the spike coupling to SLHFO phase is spurious; this effect simply reflects that filtered spike waveforms modulate their own spike timing (Scheffer-Teixeira et al., 2013). When referencing to the phase of LFP oscillations in other depths, we found that CA1 multiunit activity coupled to theta phase in all recorded layers (Fig. 9*A*, top). We did not find prominent multiunit coupling for LG, HG, or HFO phase in any layer, not even in layers where these oscillations are most prominent. On the other hand, SLHFO phase (spuriously) modulated multiunit activity at the pyramidal cell layer and dentate gyrus, although this effect was also somewhat apparent in other recording locations (Fig. 9*A*).

Finally, we constructed phase–energy plots triggered by spike times during aWK and REM sleep epochs (Fig. 9*B*; see Materials and Methods). The bottom traces in Fig. 9*B* show the spike-triggered averages of raw LFPs, which—consistent with spike–phase distributions—reveal spike timing preferentially occurring near the theta trough. The top plots show spike-triggered averages of the instantaneous amplitude of filtered LFP signals (each frequency was normalized by its maximum value to control for 1/*f*). Note the prominent SLHFO activity in the aWK plot, that is, a major increase in amplitude for a wide, unbound range of fast frequencies coinciding with the time of spiking (*t* = 0) near the theta trough (Fig. 9*B*, left). During REM sleep, SLHFO also appears at other theta phases (Fig. 9*B*, right), reflecting the bimodal distribution of spiking phases shown above (Fig. 8*B*). Interestingly, during REM sleep, theta-associated HFOs are also noticeable near the theta peak in this analysis (Fig. 9*B*, right).

## Discussion

In the present work, we characterized the patterns of fast network activity that coexist with theta oscillations in field potential recordings from the hippocampus and parietal cortex. Our results show that phase-amplitude coupling occurs between theta phase and the amplitude of multiple higher-frequency oscillations: LG, HG, and HFO. These oscillations differ from each other by frequency band, preferred theta phase, theta cycle of occurrence, and anatomic location. We also characterized spurious activity resultant from spike contamination of LFPs (SLHFO), common in cell-dense regions such as the pyramidal cell layer and dentate gyrus.

### Relation to previous findings

Different groups use different nomenclatures for fast field oscillations in the hippocampus. For instance, what we here refer to as LG and HG have also been termed “slow gamma” and “middle gamma,” respectively (Colgin et al., 2009; Belluscio et al., 2012; Schomburg et al., 2014). The terms “fast gamma” and “epsilon band” have been used for hippocampal activities >100 Hz concomitant with theta waves (Belluscio et al., 2012; Schomburg et al., 2014). However, although some groups (Scheffzük et al., 2011; Brankačk et al., 2012) have called fast gamma the same HFO activity described in the present work, the “fast gamma/epsilon band” activity reported by others may actually correspond to what we refer to as SLHFO. Namely, in Belluscio et al. (2012) and Schomburg et al. (2014), the authors report fast gamma activity occurring at the trough of the theta cycle, just where the units are most active (see also Lasztóczi and Klausberger, 2017). Moreover, this fast gamma activity exhibited a second local maximum near the theta peak during REM sleep (Belluscio et al., 2012), mirroring the changes in phase distribution displayed by the units from aWK to REM and similarly to what we have reported to occur for SLHFOs in the present work. On the other hand, the fast gamma in Scheffzük et al. (2011, 2013) and Brankačk et al. (2012) occurred after the peak of the theta wave, and its coupling to theta was most prominent during REM sleep. We prefer not calling spike contaminations of the LFP signal a type of gamma and rather reserve this term for genuine oscillatory activity. In this sense, we are not against calling theta-associated HFOs “fast gamma” as long as this term is uniquely used to refer to this rhythm. Note that this issue illustrates that defining brain oscillations solely based on frequency range is not ideal and may lead to confusion, as previously voiced elsewhere (Tort et al., 2010b; Cole and Voytek, 2017).

Hippocampal gamma oscillations have been the subject of intense research over the last decades (Bragin et al., 1995; Burchell et al., 1998; Csicsvari et al., 2003; Montgomery and Buzsáki, 2007; Colgin et al., 2009; Scheffer-Teixeira et al., 2012; Schomburg et al., 2014; Lasztóczi and Klausberger, 2014, 2016; Scheffer-Teixeira and Tort, 2016). In contrast, theta-associated HFOs have only recently been described (Scheffer-Teixeira et al., 2012; Tort et al., 2013). To try to understand why HFOs remain underreported, we first investigated whether differences in recording techniques could affect HFO detection. Although we found that electrode impedance does influence HFO detection, this influence is rather mild (Fig. 1). In fact, our results demonstrate that HFOs can be detected with different electrode types, such as thin wires used in tetrode recordings, single isolated wires of thicker diameter, and one-sided rectangular probe contacts (Fig. 1). Moreover, HFOs have also been previously detected in surface EEG recordings through steel screws placed at the dura mater over the parietal cortex (Scheffzük et al., 2011; Zhang et al., 2016). On the other hand, our results reveal that recording location and behavioral state are the two main determinants of HFO detection. During aWK, HFOs may be detected in the parietal cortex, where their amplitude is weakly coupled to theta phase (Fig. 3). During REM sleep, however, both HFO power but mainly theta-phase modulation of HFO amplitude become considerably higher, and theta–HFO coupling can also be detected in the hippocampus (Fig. 3*A*).

In our previous work (Scheffer-Teixeira et al., 2012), we used handmade electrode bundles to simultaneously record from different hippocampal layers. We showed that (1) the amplitudes of HFO and HG couple to theta phase, (2) coupling patterns depend on the hippocampal layer, (3) coupling strength is positively correlated with theta amplitude, and (4) HFO and HG have maximum amplitude in different theta phases. Here we were able to reproduce all these results in an entirely new dataset (different subjects, electrodes, recording system, and laboratory). We used commercial linear probes with a higher number of contacts, which allowed for a more fine-grained analysis of comodulation patterns and a greater span of recording locations than in our first study (Fig. 3). The results show that the recording depth in which comodulation patterns switch between theta–HFO and theta–HG coupling depends on behavioral state, a feature we had not previously recognized: during REM sleep, theta–HFO coupling is apparent down to the pyramidal cell layer, whereas during aWK, theta–HFO coupling is mostly restricted to the parietal cortex. In addition, here we were able to observe coupling between theta and LG in some recorded regions (parietal cortex and stratum radiatum), which was not detected in our previous study. Theta–LG coupling strength, however, was much weaker than theta–HG and theta–HFO coupling. Moreover, as opposed to theta–HG and theta–HFO coupling, theta–LG coupling did not increase during REM sleep.

### aWK versus REM sleep

Neuromodulatory content differs between aWK and REM sleep. During REM sleep, the hippocampus exhibits decreased serotoninergic and noradrenergic tonus and a higher cholinergic drive (Kametani and Kawamura, 1990; McCarley et al., 1995; Park et al., 1999; Graves et al., 2001; Lee et al., 2005). Theta activity during REM sleep may relate to higher acetylcholine release in the hippocampus (Kametani and Kawamura, 1990; Monmaur et al., 1997; Lee et al., 2005), which would thus characterize it as type 2 theta (Kramis et al., 1975). Here we found augmented theta amplitude during REM sleep in comparison to aWK (Fig. 5*D*), a profile similar to that evoked by cholinergic agonists (Markowska et al., 1995; Monmaur et al., 1997). Of note, the higher theta–HG and theta–HFO coupling during REM sleep are not explained by changes in theta power (Fig. 5*C–E*): during REM sleep theta power increased <1.5-fold, whereas theta–HG and theta–HFO coupling increased >10-fold. Interestingly, Newman et al. (2013) reported that scopolamine reduces theta–HG coupling in the entorhinal cortex, whereas theta–LG is unaffected. The dependence of theta–HG coupling on cholinergic transmission may be related to its increase during REM sleep; by the same token, the lack of increase in theta–LG coupling during REM sleep may be related to its insensitivity to scopolamine as observed in Newman et al. (2013). On the other hand, the dependence of theta–HFO coupling on cholinergic modulation is currently unknown. In any event, both acetylcholine and REM sleep have been associated with memory consolidation (Hasselmo, 1999, 2006; Stickgold and Walker, 2007; Nishida et al., 2009), and it is thus possible that enhanced theta–HFO and theta–HG coupling during REM sleep play functional roles.

### Coordination of fast oscillations in different theta cycles and theta phases

Bursts of fast oscillatory activity tend to happen in different theta cycles: most theta cycles containing at least one oscillatory burst were not superimposed (Fig. 7*B*). Consistent with our findings, Colgin et al. (2009) found that ∼54% of theta cycles contained at least one gamma burst, and most cycles contained just one type of gamma. In contrast, however, Belluscio et al. (2012) found that 41% of theta cycles contained both low and high gamma. Although the reason for these discrepancies is not clear, they could be due to methodological differences (for instance, our burst threshold was computed using only theta periods; they used the whole recording session) or particularities in behavior or cognitive demands between studies.

The amplitudes of LG, HG, and HFO peaked at different theta phases. HG was maximal near the theta peak and HFO soon afterward (at ∼180° and ∼210° respectively; here and below, the phases correspond to theta recorded above s. pyramidale, that is, before the phase reversal). The theta phase of maximal HG amplitude observed here is similar to that previously reported (Tort et al., 2008; Chen et al., 2011; Belluscio et al., 2012; Scheffer-Teixeira et al., 2012; Schomburg et al., 2014; Yamamoto et al., 2014; but see Colgin et al., 2009). As discussed above, the theta phase of maximal HFO amplitude is similar to that found in previous reports describing a same pattern of bounded LFP activity between 110 and 160 Hz (Tort et al., 2008; Sirota et al., 2008; Scheffzük et al., 2011; Brankačk et al., 2012; Scheffer-Teixeira et al., 2012; Tort et al., 2013; Zhang et al., 2016) but differs from other studies analyzing recordings from the pyramidal cell layer (Belluscio et al., 2012; Schomburg et al., 2014), probably because of the influence of spike contamination of the LFP signal in the latter.

Interestingly, in the parietal cortex, LG amplitude was maximal at the ascending phase of the theta cycle (∼90°), whereas in the hippocampus, LG amplitude peaked at the descending theta phase. Newman et al. (2013) found that LG recorded in the medial entorhinal cortex was maximal at the ascending phase, i.e., similar to our parietal cortex recordings. On the other hand, previous studies have reported different preferred theta phases for LG recorded in the hippocampus (e.g., Colgin et al., 2009; Yamamoto et al., 2014; Lasztóczi and Klausberger, 2017), although most of them seem to find maximal LG at the descending phase, as observed here (Chen et al., 2011; Belluscio et al., 2012; Schomburg et al., 2014; Lasztóczi and Klausberger, 2014, 2016; Lopez-Pigozzi et al., 2016). For example, Chen et al. (2011) found LG at the descending phase but with the exact phase depending on speed, Belluscio et al. (2012) found LG at the descending phase closer to the theta peak, and Schomburg et al. (2014) found LG at the descending phase closer to the theta trough. The reason for such differences is presently unclear; we suspect it may have to do with traveling wave properties (Lubenov and Siapas, 2009; Patel et al., 2012) of LFP oscillations along with differences in recording location across the antero-posterior or medio-lateral axes of the hippocampus (see Schomburg et al., 2014).

Interestingly, although hippocampal neurons spike at the trough of the theta during aWK, during REM sleep they also tend to spike more at the descending phase of the cycle after the theta peak (Fig. 8*B*; Poe et al., 2000; Mizuseki et al., 2011; Belluscio et al., 2012), where we found HFO amplitude to be maximal. Thus, HFOs may potentially be related to spiking activity at this theta phase during REM sleep. Previous work at the single-unit level has shown that some pyramidal cells shift their preferred theta phase of spiking during REM sleep (Poe et al., 2000). Furthermore, Mizuseki et al. (2011) showed that whereas nonshifting pyramidal cells are closer to stratum radiatum, cells that shift the spiking theta phase are closer to stratum oriens, that is, anatomically closer to where theta-associated HFOs are best detected in the hippocampus.

## Conclusions

In summary, here we used comodulation analyses to unveil different types of fast LFP activity in multisite recordings from the parietal cortex to the hippocampus. The results show that theta phase modulates the amplitude of three separated bands: LG, HG, and HFOs. In addition, we showed that the modulation of multiunit activity by theta phase translates into spurious modulation of a wide range of fast LFP activity, termed SLHFO, which overlaps in frequency with genuine oscillations. The bulk of these results corroborate and extend recent work of ours (Scheffer-Teixeira et al., 2012, 2013) and should help placing theta-associated HFOs as a new type of cortical oscillation (Tort et al., 2013). As such, HFOs would benefit from a thorough investigation by independent groups, as it has been the case for gamma oscillations. In this sense, we hope our results serve as a guide for identifying HFOs in other laboratories worldwide.

## Synthesis

Reviewing Editor: Liset Menendez de la Prida, Instituto Cajal CSIC Decisions are customarily a result of the Reviewing Editor and the peer reviewers coming together and discussing their recommendations until a consensus is reached. When revisions are invited, a fact-based synthesis statement explaining their decision and outlining what is needed to prepare a revision will be listed below. The following reviewer(s) agreed to reveal their identity: Guglielmo Foffani

Your ms was revised by one expert reviewer (signal processing) and me. We both agree there is value in your data. However, we both have raised several issues that you may need to address more carefully. In particular, concerns on how are groups defined and whether appropiate statistical design are used should be carefully considered. In addition, and regarding your new data on theta-HFO coupling, you need to carefully address potential discrepancies with existing data regarding theta-LG and VC issues.

I copy below the two independent reports.

R1 comments:

General comment

Using previously published data in freely moving rats, the authors offer a detailed analysis of phase-amplitude coupling between theta oscillations and oscillations at higher frequencies in the hippocampus and parietal cortex (low-gamma, high-gamma and high-frequency oscillations). They examine the dependence of phase-amplitude coupling on electrode type, recording location, behavioral state, theta phase, and spike contamination. The narrative is clear and the results are interesting and both methodologically and physiologically important. However, in several instances the study seems unpowered (n=7 animals) and some claims too strong, without sufficient attention to the variability in the data. Ideally, the authors should increase the number of animals. If increasing the number of animals is not an option, the manuscript should be reworked to clarify which results are really significant and giving more justice to biological variability.

Specific comments

1) Methods, Statistics.

The authors set high statistical standards, which is good, by their data do not seem to allow them always fulfill these standards. I totally agree that ‘p-value only’ is not sufficient, but p-value-at-least is necessary, with proper corrections for multiple comparisons (confidence-interval-only is also not sufficient).

I also fully agree that sample size should be ‘number of animals’, but this also implies that confidence intervals should be calculated using animals as samples.

The authors would probably need more animals for all their results to pass at least a ‘p-value only’ threshold.

Please add sample sizes in Table 1 (which is indeed a very useful table).

Please always fully report both significant p-values (not just p<0.05) and non-significant p-values (not just n.s. or p>0.05)

2) Results, electrode type

Please clarify number of animals and sessions.

Assuming that it is correct to use sessions as sample size (which I think it is ok in this case), Fig. 1C-right might reach significant with a t-test (sample size seems sufficiently high to reasonably respect the normality-of-errors assumption, and t-test is very robust to its violation anyway). The message would not substantially change, as the effect size in Fig. 1C left is clearly much higher than Fig. 1C right (effect size should be reported here to support the claim).

3) Results, recording location

What theta oscillation is used for the coupling? theta at the same contact where the high-frequency oscillations are recorded? at a fixed contact? (is there any difference between the two approaches?).

If theta does not come always from the same fixed contact, how much is the contribution of theta-amplitude variability between contacts to the coupling?

“theta-HFO coupling was detected above the pyramidal cell layer, while theta-HG coupling occurred below it”: the data shown in Fig. 3 seem to show a more rich and variable picture.

The transition from Fig. 3A and Fig 3C is somewhat confusing due to the different scaling; I suggest the color code to be the same for all plots, in order to better compare the strength of the comodulation at different depths/frequencies/states.

Apart from the side result on delta oscillations (which is admittedly outside the scope of the present work), the depth comodulogram are not particularly informative, in the sense that an x-y plot with depth on the x-axis and amplitude of the modulation index between theta and the corresponding band on the y-axis would be more clear.

Related to the above, the authors are not reporting the potentially interesting variability in the frequency of the high-frequency oscillations (and of gamma) in the coupling, which is evident in Fig. 3A: depth comodulograms with the frequency of the amplitude-modulated oscillation in the x-axis would be very informative.

“during aWK, theta-HFO coupling may only be detected in the parietal cortex”: this statement seems too strong and might be toned down to better reflect the variability in the data.

4) Results, behavioral state

There is redundancy with the previous section, which makes the narrative of the transition a little confusing.

All the results of this section should be supported by p-values (i.e. proper ANOVAs), using animals as sample size.

5) Results, theta phase

Exact p-values should be given. Please ensure that results are properly corrected for multiple comparisons.

Figure 5B. With n=7 animals, I suggest to find a more explicit representation to better convey the variability in the data.

Errors in the text seem very small: what are these numbers? SD? SE?

6) Results, spike contamination

“We filtered LFPs above 150Hz to identify SLHFOs while avoiding capturing genuine HFOs”. Why is the SLHFOs band overlapping with HFO? Please clarify.

7) Discussion

The authors should revise the discussion after confirming what results hold after the new statistical analyses and tone down some claims to better reflect biological variability.

------------------------

Editor comments

There is some discrepancy with published hippocampal data (Buzsaki, Klausberger, Mehta and our own observations in Lopez-Pigozzi et al. eNeuro 2016) requiring clarification for findings on HFO to be integrated in the existing framework. Specifically hippocampal theta-LG (30-60 Hz) has been consistently found at the falling theta phase (with respect to CA1 stratum pyramidale) in all these laboratories independently. However, your data show LG occurring at the ascending theta phase (Fig.5). While speed and proximodistal recording location may influence LG dynamics (closer or not to trough and peaks), its phase preference remains relatively restricted (consistent with the arrival of CA3 inputs to SR and firing specificity of PV-basket cells being activated feedforward by CA3). What channel is shown in Fig.5? Is that the case of the parietal cortex? Please, show phase information for parietal and hippocampal layers separately and clarify. In the same vein, please specify data origin in every analysis shown (e.g. what channels are shown in Fig.1D).

LFP signals can suffer from volume conduction and summation of different generators. Several methods can be applied for adequate separation, from CSD to factor analysis. In our CSD analysis for instance, we found that phase-locked activity faster than 90 Hz cannot be adequately separated from firing contribution in hippocampal layers (see Lopez-Pigozzi et al. eNeuro). Given authors have access to laminar LFP profiles, the ms would benefit from some analysis to separate local generators.

To facilitate understanding please, specify phase preference (and location) for each frequency band in the abstract. Check consistency of frequency bands across the ms (theta 5-10 Hz vs 6-10 Hz).

Raw traces are important to illustrate the phenomena and to facilitate comparison across laboratories. This is critical to support HFOs are genuine LFP oscillations, but also for appreciating dynamics, phase and location preference of LG, HG and HFOs. Please, add raw traces to Fig.5.

Are implanting coordinated histologically validated? Please, specify in Methods. The image in Fig.2A shows poor correspondence with LFP recording at 2B. Hippocampal ripples expands 100-200 um at most around CA1 str pyramidale but here they seem to spread wide (assuming 100 um separation between sites; please specify probe design in Methods). Some of these profiles are more typical of distal locations where the CA1 stratum pyramidale become wider.

Where is reference used for recording? Please, specify in Methods

Theta cycles are asymmetric. At SLM they are maximal in amplitude and asymmetry, at SP there are some inconsistencies in the definition. Please clarify how are theta cycle timing identified from 4-20 Hz band (what channel)?

Contribution by MUA and spike-leaked HFOs may evolve along days of implantation. Did you noted any trend?

Comparison between electrode types need to specify shape (wire vs planar) and material (tungsten vs Pt-Ir vs silicon). Note tissue reaction occur all along the shank more than at the electrode tip, which for silicon probes may differentially affect sensing sites along the probe. Please, specify what region is data shown in Fig.1

Results, line 261-270: Fig.3A doesn't clearly show what is intended. Useful to add a line at zero depth in Fig.1C

Introduction, line 47: LG is believed to originate from CA3 neurons synapsing on CA1 pyr cells AND INTERNEURONS.

AE comments:
